# Self-Referencing Photothermal Common-Path Interferometry to Measure Absorption of Si_3_N_4_ Membranes for Laser-Light Sails

**DOI:** 10.1021/acsphotonics.5c01886

**Published:** 2025-10-23

**Authors:** Tanuj Kumar, Demeng Feng, Shenwei Yin, Merlin Mah, Phyo Lin, Margaret A. Fortman, Gabriel R. Jaffe, Chenghao Wan, Hongyan Mei, Yuzhe Xiao, Ron Synowicki, Ronald J. Warzoha, Victor W. Brar, Joseph J. Talghader, Mikhail A. Kats

**Affiliations:** † Department of Electrical and Computer Engineering, 5228 University of Wisconsin—Madison, Madison, Wisconsin 53706, United States; ‡ Department of Electrical and Computer Engineering, 5635 University of Minnesota—Twin Cities, Minneapolis, Minnesota 55455, United States; § Department of Physics, University of Wisconsin—Madison, Madison, Wisconsin 53706, United States; ∥ Department of Electrical Engineering, Stanford University, Stanford, California 94305, United States; ⊥ Department of Physics, University of North Texas, Denton, Texas 76203, United States; # 481925 J. A. Woollam Co. Inc., 645 M St Suite 102, Lincoln, Nebraska 68508, United States; ∇ Department of Mechanical Engineering, 32722 United States Naval Academy, Annapolis, Maryland 21402, United States

**Keywords:** ultralow optical loss measurement, photothermal common-path interferometry, silicon nitride, laser sails, graphene

## Abstract

Laser-light sails are a spacecraft concept, wherein lightweight “sails” are propelled by high-intensity lasers. We investigated the near-infrared absorption of free-standing membranes of stoichiometric silicon nitride (Si_3_N_4_), a candidate sail material. To resolve the small but nonzero optical loss, we used photothermal common-path interferometry (PCI), for which we developed a self-referencing modality where a PCI measurement is performed twice: once on a bare membrane, and a second time with monolayer graphene deposited on the membrane. The graphene increases the absorption of the sample by orders of magnitude, such that it can be measured by ellipsometry without significantly affecting the thermal properties. We measured the absorption coefficient of Si_3_N_4_ to be (1.5–3) × 10^–2^ cm^–1^ at 1064 nm, making it a suitable sail material for laser intensities as high as ∼10 GW/m^2^. By comparison, silicon-rich “low stress” SiN_*x*_ (*x* ∼ 1), with a measured absorption coefficient of approximately 8 cm^–1^, is unlikely to survive such high laser intensities. Our self-referencing technique enables the testing of low-loss membranes of various materials for laser sails and other applications.

## Graphical abstract

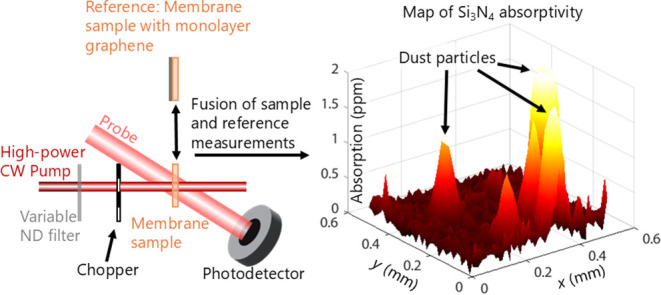

## Introduction

Precise measurement of optical absorption in low-loss materials is important for applications from on-chip photonics to sensitive experiments like gravitational-wave detection in LIGO.^1–4^ An application of recent interest is the development of light sails propelled by high-power lasers, where laser intensities as high as 10–100 GW/m^2^ are being considered.^5–7^

The choice of materials is important to achieve efficient acceleration and maintain sail integrity under intense illumination, with requirements that include low linear and nonlinear absorption, high refractive index (to maximize reflectivity with the smallest amount of material^8,9^ ), low mass density, and high thermal conductivity.^9–11^ Stoichiometric silicon nitride (Si_3_N_4_) is being considered for laser-sail applications due to its moderately high refractive index (∼2), low loss in the near-infrared, large band gap of at least ∼3.3 eV^12,13^ that is inconducive to near-infrared two-photon absorption, and high extinction coefficient in the mid-infrared, which can aid in radiative cooling.^8^

Measuring precise values of the absorption coefficients of low-loss materials, such as Si_3_N_4_ in the near-infrared, is challenging for the same reason that it is useful (i.e., because the losses are low), and conventional techniques such as ellipsometry and reflection/transmission spectroscopy can be insufficient. This is especially the case for samples with a membrane form factor, such as for light sails. There have been several measurements of Si_3_N_4_ using cavity ring-down spectroscopy with microfabricated waveguide resonators,^1,14,15^ but these measurements may not be directly applicable for suspended membranes in free space due to the potential presence of scattering losses and other interface effects that are difficult to distinguish from absorption losses, as well as due to differences in material strain. There is therefore a need for direct measurement of the optical absorption of membranes of Si_3_N_4_ and other low-loss materials, such as certain layered van der Waals materials.^16–18^

We explore photothermal common-path interferometry (PCI)^2,19–22^ to directly measure the optical absorption of suspended low-loss membranes. In PCI, a chopped continuous-wave pump laser is incident on the material being tested, resulting in heating. The small increase in temperature results in a change of refractive index via the thermo-optic effect, and this change is measured using a probe laser at a different wavelength and incident angle compared to the pump laser.^2,3,22–24^ The conversion from a PCI measurement to an absolute absorption value is not trivial because it is a function of both optical and thermal processes, and we found that most methods in the literature^2,20–26^ are difficult to use for freestanding structures that have nontrivial thermal conduction to the supporting frame.

We present a new self-referencing PCI modality, in which a PCI measurement is performed on a suspended membrane of interest and then on an identical sample onto which we have transferred a monolayer of graphene. Monolayer graphene has a well-known and large optical absorption (∼2.3% in free space), which is readily measurable by conventional optical techniques,^27–29^ while the thermal conductance of supported graphene is modest due to its monolayer thickness and the suppression of in-plane phonon transport,^30–32^ compared to suspended graphene.^33,34^ The addition of graphene dramatically increases the optical absorption to values measurable using conventional techniques, while not changing the thermal properties very much, thus serving as an ideal reference sample for the PCI measurement.

Using our self-referencing PCI technique, we measured the absorption coefficients of stoichiometric Si_3_N_4_ and silicon-rich SiN_*x*_ (*x* ∼ 1), determining that Si_3_N_4_ may be suitable for laser sails with laser intensities approaching ∼10 GW/m^2^. Our self-referencing PCI technique enables the direct measurement of absorptivity in low-loss membranes without having to account for substrate effects.

## Self-Referencing Photothermal Common-Path Interferometry

### Photothermal Common-Path Interferometry (PCI)

PCI measures the perturbation of a probe beam passing through a region of localized thermal lensing created by the absorption of a high-powered, chopped, continuous-wave pump laser (Figure 1a).^4,22,35^ The small temporally periodic increase in temperature in that region results in a change of refractive index via the thermo-optic effect, creating a thermal-lensing effect, and this change is measured using a probe laser at a different wavelength that is at an angle to the pump (Figure 1a).^2,3,19,22–24^ Since the probe laser is bigger in diameter than the pump, the perturbed and unperturbed parts of the probe interfere, leading to a diffraction pattern in the detector plane.^4,19,22^ The intensity of the central peak of the diffraction pattern carries information about the absorption of the material. Because the pump is chopped, the signal at the detector (which measures the peak of the diffraction pattern) consists of alternating current (AC) and direct current (DC) components (*V*_AC_ and *V*_DC_ respectively). There is a delay, or phase difference, between the chopper and measured AC signal at the detector, which is related to the time constant of thermal dissipation (Figure 1b).^2,22^ The peak *V*_AC_ for a sample is used in calculations, which is achieved when the waists of both the pump and the probe are at the sample surface. To obtain this laser–sample configuration, the sample is moved in the *z*-direction (along the pump beam) until the characteristic peak in *V*_AC_ is observed.

**Figure 1:**
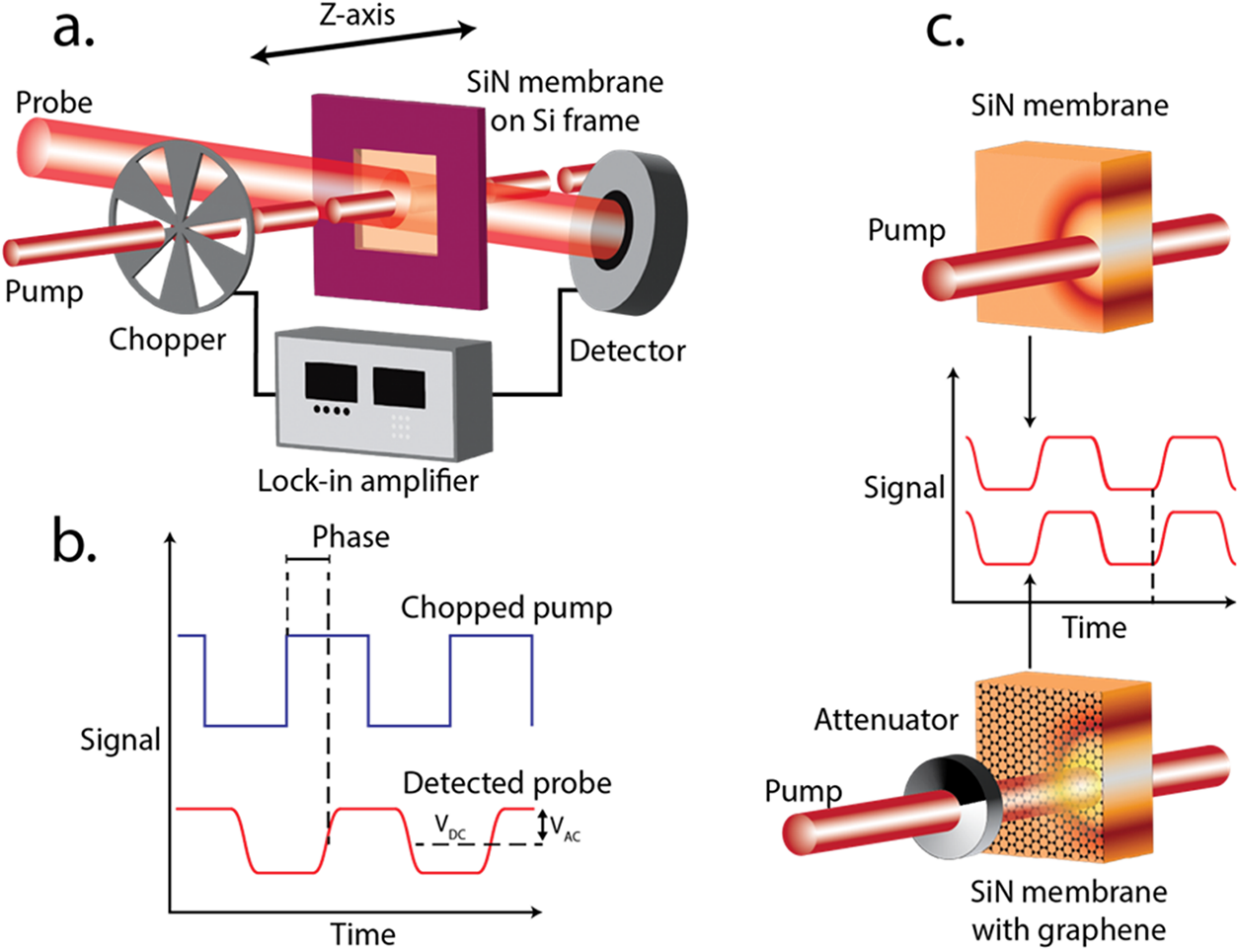
(a) Schematic of the photothermal common-path interferometry (PCI) setup, along with (b) a visualization of the PCI signal (with AC and DC components) and phase resulting from the time delay between chopped light and detected probe intensity. (c) Transfer of a graphene monolayer onto a sample to increase optical absorption. The measurement of absorption then involves attenuating the pump until the same value of PCI signal is measured with graphene as the unattenuated measurement without graphene. In our experiments with silicon nitride membranes, the addition of graphene did not significantly alter the PCI phase, indicating that the thermal conductance of the sample was not significantly altered.

A PCI measurement does not directly provide the absolute absorptivity value; instead, the absorptivity *A* (a unitless number between 0 and 1) must be obtained by translating from the observed PCI signal. To first order, the absorptivity of the sample, *A*, can be related to *V*_AC_, *V*_DC_, and *P*_pump_ by^4,35^

1 $$A=\frac{K\cdot V_{\mathrm{AC}}}{{P_{\mathrm{pump}}V}_{\mathrm{DC}}}$$

where *P*_pump_ is the power of the pump beam, and *K* is sometimes referred to as a calibration or correction factor. The definition of *K* and the form of eq 1 can vary.^4,19–21,35^ In the present paper, *K* has units of Watts, but there are certain works where both sides of eq 1 have been normalized by sample thickness.^4,21^ *K* can depend on many variables, including the crossing angle, wavelength, and shape and size of the laser beams^21–24^ and the sample’s geometry and thermal properties, which include the heat capacity, thermal conductivity, thermo-optic coefficient, and coefficient of thermal expansion. Note that thermal expansion affects the PCI signal via deformation in addition to the thermo-optic effect.^22^ Therefore, *K* must be determined for every new laser-beam setup, material, and geometry.

There exist various ways to determine *K* in the literature, but we found them challenging to apply to membranes. In one way to find *K*, a thin film of the sample in question can be grown on or transferred to a fused-silica substrate^2,21,25,26^ or another substrate for which *K* is known.^21^ Si_3_N_4_ growth is a nontrivial process requiring optimization of parameters such as gas flow, pressure, and temperature.^25,36^ In addition, the form factor of a film on a substrate cuts off access to the back side of the film/membrane, which may be needed for future experiments such as the impact of dust on light sails.^11^ We note that the approach involving film growth on a known substrate only works for films with a thickness of <1–10 μm because thermal lensing in thicker films (as opposed to the substrate underneath) can modify the PCI signal.^2,21,35^ Another approach to determine *K* is to perform a PCI measurement at a substantially different wavelength, where the optical absorptivity is larger and can be measured independently. However, this requires keeping the pump shape and size the same across different wavelengths.^20,21^ *K* has also been calculated theoretically,^21,23,24^ but the required multiphysics simulations have many input parameters, resulting in many potential sources of error. A table of various methods to determine *K* in the literature is available in Supporting Information S1.

### Self-Referencing Technique for PCI

Our new self-referencing PCI technique to calculate *K* follows the philosophy that the PCI reference sample should be as similar as possible to the sample being tested.^22^ In this modality, we perform two PCI measurements: the first with the sample being investigated and the second with the same (or identical) sample with a graphene monolayer transferred onto it. The use of monolayer graphene enhances the optical absorptivity of this reference sample to levels measurable by methods simpler than PCI (e.g., ellipsometry), while leaving its thermal conductance mostly unchanged, enabling the calculation of *K* for a suspended membrane sample using eq 1. The thermal conductance is further discussed later in this manuscript.

To prepare the reference sample, we transferred chemical vapour deposition (CVD)-grown monolayer graphene onto Si_3_N_4_ and SiN_*x*_ membranes purchased from Norcada Inc.^37^ (see Supporting Information S2 for membrane geometry) and then measured the absorptivity (*A*_ref_) using variable-angle spectroscopic ellipsometry (see Supporting Information S3, S4, and S8) to be 1.5 ± 0.11% for the ∼194 nm thick Si_3_N_4_ and 2.6 ± 0.16% for the ∼2-μm thick SiN_*x*_ at 1064 nm. These numbers are different from the well-known ∼2.3% absorptivity for suspended graphene^27–29^ due to Fabry–Pérot effects. Precise thicknesses of the membranes were also calculated from ellipsometric measurements. Any impurities that may have been introduced during the transfer of graphene would also be accounted for in our ellipsometric measurements.

Then, we performed PCI measurements on silicon nitride membranes with and without graphene. For each PCI measurement, we translated the sample position along the *z*-axis (Figure 2a) and recorded the PCI signal. The position where the PCI signal reaches its maximum (peak positions in Figure 2b for SiN_*x*_ and Figure 2c for Si_3_N_4_ (data set 1)) corresponds to the sample position where the pump and probe beams cross at the membrane; the values of *V*_AC_ and phase at this position of maximal signal are then used for further analysis. We used a sufficiently powerful continuous-wave pump laser to obtain measurable PCI signals (*V*_AC_, *V*_DC_, and the phase) for the samples without graphene (*P*_pump,sample_ = ∼2 W for Si_3_N_4_ and ∼250 mW for SiN_*x*_) and then attenuated the pump laser by many orders of magnitude (*P*_pump,ref_ = ∼45 μW for Si_3_N_4_, and ∼20 mW for SiN_*x*_) to achieve similar *V*_AC_ values between the sample and its graphene-coated reference.

**Figure 2:**
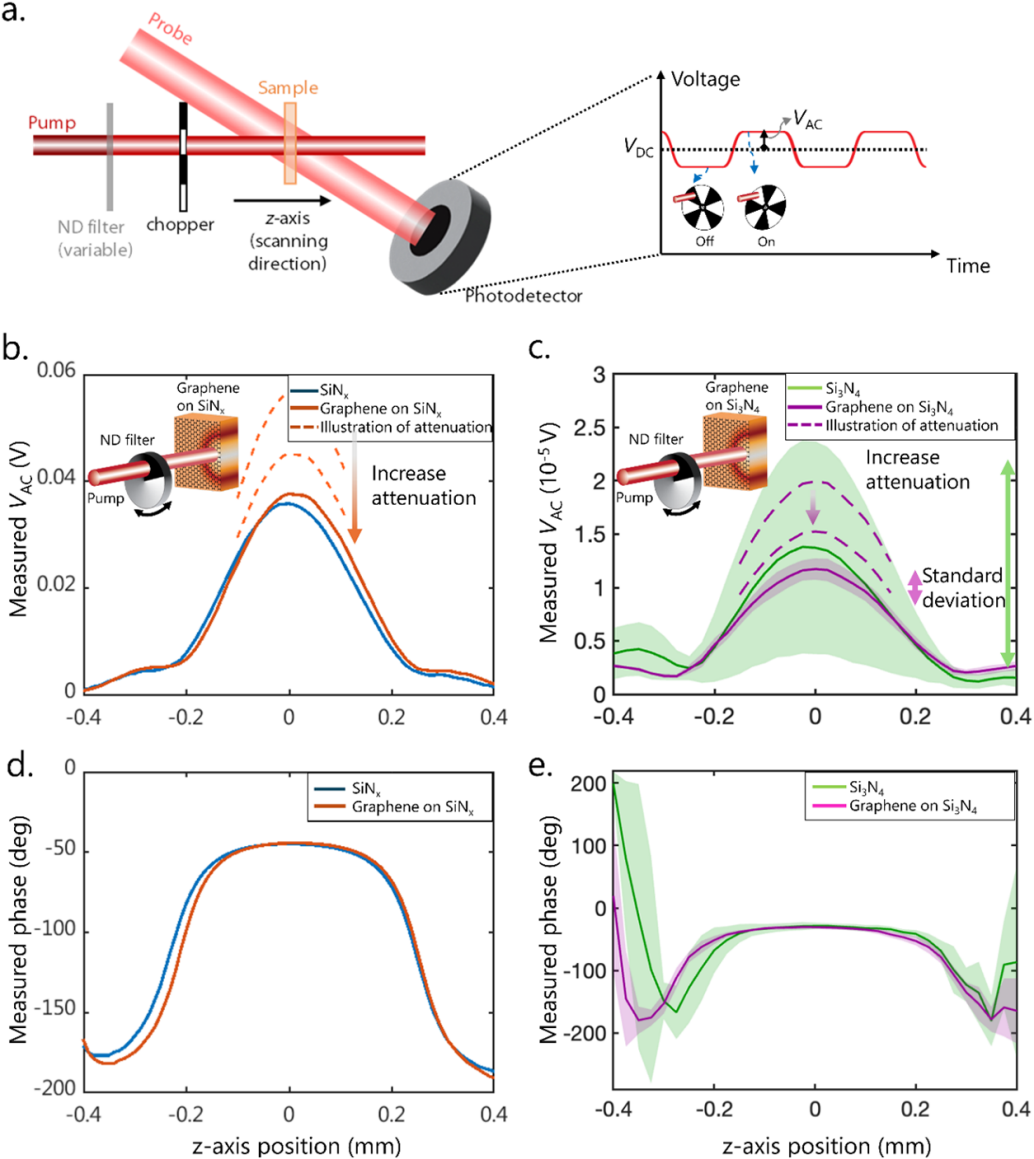
(a) Side-view schematic of the PCI setup showing translation of the sample along the *z*-axis, and the AC and DC components of the detected signal. The sample is translated in the *z*-direction to find the peak of the AC signal, which occurs when the pump waist is at the sample surface; (b) AC component of the detected probe intensity (*V*_AC_) for the SiN_*x*_ membrane with and without graphene. The pump intensity was manually attenuated using a variable ND-filter for the sample with graphene to obtain a *V*_AC_ similar *V*_AC_ to that of SiN_*x*_ alone (inset). Solid lines are the measured *V*_AC_, while dashed lines represent the process of increasing attenuation to achieve a similar *V*_AC_ with and without graphene. Because of this manual attenuation process over many orders of magnitude of pump power, it was possible to bring the *V*_AC_ of graphene-on-SiN_*x*_ very close to that of SiN_*x*_, but a slight difference in *V*_AC_ remained. That difference can be addressed via eq 1 when calculating the absorption. (c) *V*_AC_ for the Si_3_N_4_ membrane and Si_3_N_4_ with graphene (data set 1), similarly obtained using a variable ND-filter. Due to the low loss of Si_3_N_4_, measurements spanned two data sets, and the average (solid lines) and standard deviation (shaded areas) of five measurements taken for Si_3_N_4_ and Si_3_N_4_ with graphene (data set 1) are shown. Dashed lines illustrate the process of attenuation and do not represent actual measured data. (d, e) Phase between the chopped pump and detected probe intensities vs the sample position for (d) the SiN_*x*_ membrane and (e) Si_3_N_4_ membrane with and without graphene (data set 1).

Because the absorptivity of the graphene-coated samples (*A*_ref_) was already measured using ellipsometry, we used our PCI measurements and eq 1 to calculate *K* and then used *K* to obtain absorptivities for the Si_3_N_4_ and SiN_*x*_ samples. We performed one set of measurements for SiN_*x*_ (Figures 2b,d and 3b), but two sets of measurements for the low-loss Si_3_N_4_: data set 1 in Figure 2c,e and data set 2 in Figure 3a and Supporting Information. Si_3_N_4_ data set 1 comprises data with high SNR but at fewer points on the membrane, while Si_3_N_4_ data set 2 comprises data with lower SNR spanning many more points as shown in Figure 3a.

**Figure 3:**
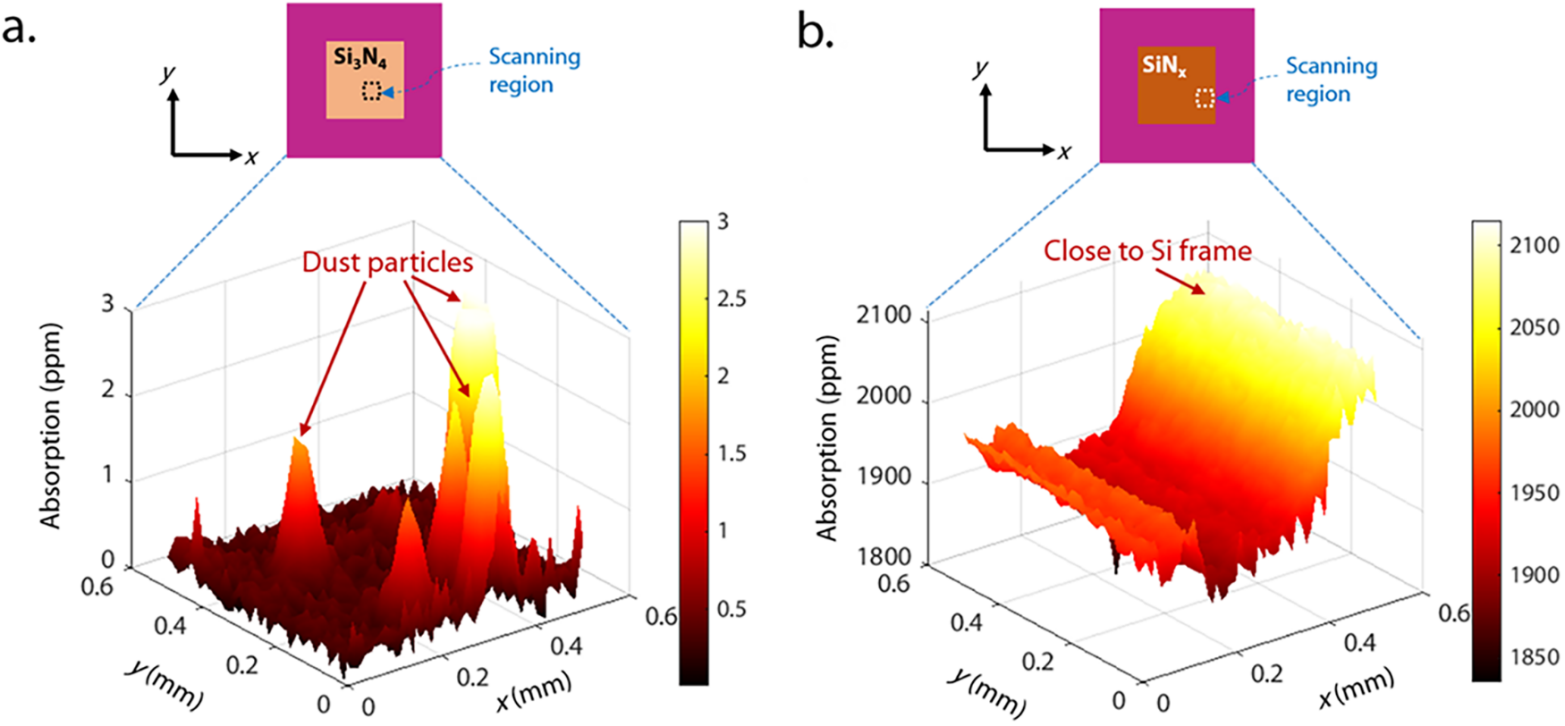
Two-dimensional (2D) scans of absorptivity for (a) Si_3_N_4_ (data set 2) and (b) SiN_*x*_ (*x* ∼ 1) membranes, in parts per million (ppm). The scanned area is a 0.5 × 0.5 mm^2^ (dashed box on the membrane schematic) and visually presents spatial variability in absorption in the membranes. For the Si_3_N_4_ membrane, the sharp absorptivity peaks correspond to dust on or sample defects in the membrane, presenting a possible challenge for future laser sails. For the SiN_*x*_ membrane, the measured absorption increases as the pump beam spot approaches the boundary of the membrane such that a portion of the pump is absorbed in the Si frame. More information about the membrane dimensions is available in Supporting Information S2.

## Results

We took five absorptivity measurements on Si_3_N_4_ (data set 1), with results ranging from 2.47 × 10^–7^ to 17 × 10^–7^, corresponding to absorption coefficients from 1.53 × 10^–2^ to 10.56 × 10^–2^ cm^–1^. On Si_3_N_4_ (data set 2), we conducted a scan over 2601 points spanning an area of 0.5 mm × 0.5 mm, as shown in Figure 3a. We observed spikes in absorptivity that we believe to be dust or sample defects,^2,21^ which were disregarded for the calculation of average absorptivity. A study to identify the origin of the spikes as either dust or defects is beyond the scope of this work, although we did observe a spike present itself in real time during an experiment, pointing toward a dust particle landing on the sample. The size of the spikes is ∼80 μm, close to the pump diameter of 70 μm, implying that the dust particles or sample defects are potentially much smaller than 80 μm. After discarding the outliers, we were left with ∼1600 different spatial points, with the average absorptivity equal to (3.4 ± 1.2) × 10^–7^ in the ∼194 nm membrane (i.e., Si_3_N_4_ (data set 2)). Using the transfer-matrix method (see Supporting Information S5), we converted this value to the absorption coefficient (2.09 ± 0.76) × 10^–2^ cm^–1^ at 1064 nm. Because the number of measurements and quality of data were different between Si_3_N_4_ data sets 1 and 2, we are unable to report an estimate for the absorption coefficient with an error bar. Nevertheless, based on the two sets of data, we estimate that the absorption coefficient of Si_3_N_4_ is between 1.5 × 10^–2^ and 2.9 × 10^–2^ cm^–1^.

For comparison, Land et al.^38^ conducted nanomechanical absorption spectroscopy and reported an Si_3_N_4_ extinction coefficient (κ) of 1.8 × 10^–7^ at 1064 nm, which corresponds to an absorption coefficient of 2.13 × 10^–2^ cm^–1^. At 1550 nm, Ji et al. and Luke et al. reported absorption coefficients of 3 × 10^–4^ and 6.8 × 10^–3^ cm^–1^, respectively, using cavity ring-down spectroscopy, which involves separating other loss mechanisms such as scattering. We expect lower absorptivity at 1550 nm compared to that at 1064 nm because there are no resonances until the mid-IR, though we also note (to our surprise) that Land et al. reported a higher absorption coefficient of 6 × 10^–2^ cm^–1^ at 1550 nm.

For the SiN_*x*_ membrane, we conducted measurements over 2601 points spanning an area of 0.5 mm × 0.5 mm, as we did with Si_3_N_4_ (data set 2). We observed an increase in absorptivity when the pump laser beam was close to the Si frame (Figure 3b) and excluded these points from the average absorptivity calculation (approximately 1/3 points removed, see histogram in Supporting Information S5). We calculated the average absorptivity to be (1.94 ± 0.12) × 10^–3^ for the ∼2-μm SiN_*x*_ membrane, corresponding to an absorption coefficient of 7.94 ± 0.50 cm^–1^ (Supporting Information S5). This is close to the reported value of 6.9 ± 0.7 cm^–1^ using PCI and cavity round-trip measurements by Steinlechner et al.^3^ and is on the same order of magnitude of loss reported for various stoichiometries of PECVD-grown SiN_*x*_H_*y*_ measured using PCI.^39^ We note that in Steinlechner et al.,^3^ this number is reported for “Low-stress 2 μm Si_3_N_4_ membranes,” which we understand to actually be SiN_*x*_ membranes similar to the ones we study here (*x* ∼ 1).

## Discussion

Figure 3 demonstrates potential challenges for laser sails relating to the variability of absorption across the membrane due to both intrinsic differences in the material and increases in absorptivity caused by dust on the membrane (either due to contamination during fabrication or from space itself). The effect of space dust, and absorption variability more broadly, has been previously considered in the context of laser sails.^11^ In terms of the integrity of a hypothetical laser sail, the low absorptivity of stoichiometric Si_3_N_4_ is encouraging, though additional measurements are needed to characterize the temperature dependence of the absorption coefficient relevant in thermal runaway processes,^10^ and care must be taken to avoid variations in silicon nitride stoichiometry that lead to higher absorptivity. High-temperature measurements for these membranes are nontrivial and are beyond the scope of this work, so we perform thermal equilibrium calculations for a simple Si_3_N_4_ membrane sail under 10 GW/m^2^ laser illumination, assuming temperature-independent absorptivity. These calculations yield equilibrium temperatures from ∼700 to ∼950 K (Supporting S7), lower than the decomposition temperature of Si_3_N_4_ at 1500–1900 K.^40,41^

### Validity of the Self-Referencing Technique

A key assumption in our self-referencing PCI technique is that the pump-beam-induced thermal lensing within the sample is similar to that within the reference. This assumption can be validated because the PCI phase depends on the material’s thermal properties and is thus a good method of comparing thermal lensing between samples.^2,21^ In our self-referenced PCI experiments, the addition of graphene to a sample did not significantly change the measured PCI phase (Figure 2d,e), indicating that the heat generated during optical absorption at the graphene is quickly transferred to the sample underneath, and the overall thermal conductance is dominated by the sample itself, with only a minor contribution from the graphene or any impurities (if present) introduced during the transfer of graphene.

This observation is supported by individually considering the thermal conductances (which depend on sample thicknesses and thermal conductivities) of graphene and silicon nitride membranes. We measured the in-plane thermal conductivity of Si_3_N_4_ and SiN_*x*_ to be approximately 18 and 10.3 W/m/K, respectively, using frequency-domain thermoreflectance (see Supporting S8). We did not measure the thermal conductivity of our graphene directly, but we do not expect it to be more than roughly 1000 W/m/K, an estimate based on reported thermal conductivities of supported graphene in the literature.^30–32^ We note that the thermal conductivity of supported graphene is much lower than that of freestanding graphene (up to 5000 W/m/K) for two reasons: (a) suppression of flexural modes that are present in freestanding graphene,^31,32^ and (b) leakage of phonons across the graphene-membrane interface.^30,31^ Thermal conductance is then directly proportional to the product of thermal conductivity and sample thickness; in this case, the monolayer thickness of graphene (0.335 nm) leads to an order of magnitude lower thermal conductance (proportional to 0.3 nm × 1000 W/m/K = 3 × 10^–7^ W/K) compared to the conductance of the Si_3_N_4_ membrane (proportional to 200 nm × 18 W/m/K = 3.6 × 10^–6^ W/K). This also indicates that self-referencing PCI may not be applicable for samples with in-plane thermal conductance comparable to or lower than that of supported graphene, such as much thinner films.

## Conclusions

Characterization of optical absorption of low-loss materials is important for on-chip photonics, optical components in sensitive experiments, and (most relevant to this paper) laser-light sails. We demonstrated a self-referencing approach to photothermal common-path interferometry (PCI), wherein the transfer of monolayer graphene onto a low-loss sample significantly increases its absorptivity to create a reference for the PCI technique. For all membranes we studied, the addition of graphene did not significantly affect the thermal properties of the sample, preserving the validity of PCI. Based on two sets of measurements, we estimated the absorption coefficient of stoichiometric Si_3_N_4_ to be roughly between 1.5 × 10^–2^ and 2.9 × 10^–2^ cm^–1^ at 1064 nm. For a nonstoichiometric silicon nitride (SiN_*x*_) sample, we measured the absorption coefficient to be approximately 8 cm^–1^. The absorption coefficient of stoichiometric Si_3_N_4_ is sufficiently small to enable light sails at incident intensities approaching ∼10 GW/m^2^ assuming no runaway thermal processes, although care must be taken to avoid variations in its stoichiometry. Our self-referencing PCI technique using monolayer graphene can be applied to most suspended membranes or more-complex structures and is a promising way to evaluate low-loss materials.

## Supplementary Material



## Notes

Comparison with other PCI methods, ellipsometry of membranes with and without graphene, PCI error bar calculation and quality of data, comparison of measured Si_3_N_4_ loss with other works, application in light sails, summary of experimental methods (PDF)
