# Supplementary material for: Self-Referencing Photothermal Common-Path Interferometry to Measure Absorption of Si3N4 Membranes for Laser-Light Sails
Source: ACS Photonics. 2025 Oct 23;12(11):6381–7. doi: 10.1021/acsphotonics.5c01886 (PMC12636069; doi:10.1021/acsphotonics.5c01886)
Supplement: Supplementary file 1 [file ph5c01886_si_001.pdf]

Supplemental information for

Self-referencing photothermal common-path interferometry to measure absorption of Si_3N_4 membranes for laser-light sails

Tanuj Kumar[1‡], Demeng Feng[1‡], Shenwei Yin[1], Merlin Mah[2], Phyo Lin[2], Margaret A. Fortman[3], Gabriel R. Jaffe[3], Chenghao Wan[1,4], Hongyan Mei[1], Yuzhe Xiao[1,5], Ron Synowicki[6], Ronald J. Warzoha[7], Victor W. Brar[3], Joseph J. Talghader[2], Mikhail A. Kats[1♦]

[1]Department of Electrical and Computer Engineering, University of Wisconsin–Madison, Madison, WI 53706, USA

[2]Department of Electrical and Computer Engineering, University of Minnesota–Twin Cities, MN 55455, USA

[3]Department of Physics, University of Wisconsin–Madison, Madison, WI 53706, USA

[4]Department of Electrical Engineering, Stanford University, Stanford, CA 94305, USA

[5]Department of Physics, University of North Texas, Denton, TX, 76203, USA

[6]J. A. Woollam Co. Inc., 645 M St Suite 102, Lincoln, NE 68508, USA

[7]Department of Mechanical Engineering, United States Naval Academy, Annapolis, MD 21402, USA

‡ - equal contribution

♦ - corresponding author. Email: mkats@wisc.edu

S1. Summary of methods to determine the "correction factor" *K* in the literature

Table S1. Comparison of methods to determine K in the literature

**Method**	**Theoretical/ Experimental**	**Sample used**	**Notes**	**References (supplementary)**
Model heat distribution and probe distortion	Theoretical	Suprasil 311, KU-1	Complicated, many variables to take care of	[S1,S2]
Model probe distortion then (semi) numerically solve	Theoretical	AlGaAs on fused silica, AlGaAs on sapphire		[S3]
Grow thin-film on bulk fused silica	Experimental	AlGaAs film on fused silica	Not applicable to membranes	[S4–S7]
PCI measurement with known loss at different wavelength	Experimental	GaAs/AlGaAs coating on fused silica, Germanium	Beam shape and size must be same at shorter wavelength. Loss at different wavelength not always known	[S3,S8]
Increase loss by doping	Experimental	$LiNbO_3$		[S9]
Reference sample by addition of monolayer graphene	Experimental	Si_3N_4/SiN_x (x~1) membranes	Reference sample has similar thermal properties as but orders-of-magnitude higher absorbance than sample being tested, which bypasses sample and setup variabilities	This work

S2. Membrane geometry

We obtained ~200 nm thick Si_3N_4 and ~2 μm thick silicon-rich SiN_x ($x \sim 1$) membranes from Norcada Inc. (Edmonton, AB, Canada). Both membranes were obtained mounted on 200 μm thick, 10 mm $\times$ 10 mm Si frames, with a 5 mm $\times$ 5 mm area of suspended membrane in the middle (**Fig. S1**).

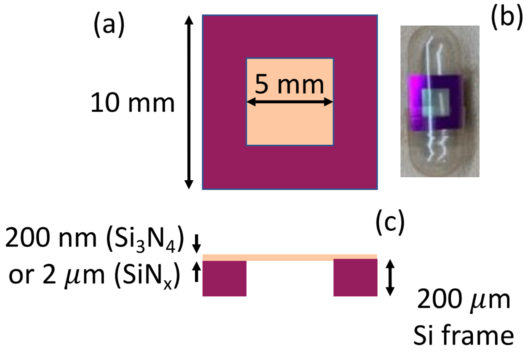

Fig. S1. (a) Front-view schematic of the membranes, (b) front-view picture of a Si_3N_4 membrane enclosed in a clear capsule, (c) side-view schematic of the membranes showing the suspended part of the membranes in the middle.

S3. Characterization of thickness and refractive index of the Si_3N_4 membrane via variable-angle spectroscopic ellipsometry

We performed variable-angle spectroscopic ellipsometry measurements on Si_3N_4 and SiN_x membranes mentioned in the main text, over a wavelength range of 300 – 1500 nm. We used a J. A. Woollam V-VASE ellipsometer for measurements, and used several different oscillator models to fit the experimental data. In this section, we discuss in detail the data and analysis for Si_3N_4, but similar analysis and conclusions apply to SiN_x ($x \sim 1$).

We used three different models to fit the ellipsometry data: Cauchy model with Urbach tail, Tauc-Lorentz model, and Cody-Lorentz model, of which the last 2 satisfy the Kramers-Kronig relations. We used the model expressions for these oscillator models from J.A. Woollam, Inc.'s handbook on using WVASE ellipsometry fitting software[S10]. The expressions, together with the resulting fitting parameters, including the thicknesses, are shown in **Table S2**. All those models fit the experimental ellipsometry data (300 – 1500 nm) well (as shown in **Fig. S2**).

Table S2. Fitted expressions of the complex relative permittivity, and the resulting membrane thickness from different models

Model	Complex relative permittivity ϵ_r [a]	Membrane thickness
Cauchy	$n = A + \frac{B}{\lambda^2} + \frac{C}{\lambda^4}$ [b]<br>$\kappa = \alpha \cdot \exp(\beta(E - \gamma))$ [c]<br>$\epsilon_r = (n + i \cdot \kappa)^2$ [d]<br>where $A = 1.9906, B = 0.014928, C = 0.0004, \alpha = 0.27783, \beta = 1.6526, \gamma = 6.199$.	194.3 nm

Tauc-Lorentz	$\epsilon_2(E) = \begin{cases} \frac{1}{E} \cdot \frac{AE_oC(E-E_g)^2}{(E^2-E_o^2)^2+C^2E^2} & (E \geq E_g) \\ 0 & (E < E_g) \end{cases}$<br>$\epsilon_1(E) = 1 + \frac{A_p}{E_p^2-E^2} + \frac{2}{\pi}\mathcal{P}\int_0^\infty \frac{\xi\epsilon_2(\xi)}{\xi^2-E^2}d\xi$ [e]<br>$\epsilon_r = \epsilon_1 + i \cdot \epsilon_2$<br>where $A = 0.69751, E_o = 5.7188, C = 0.64145, E_g = 0, A_p = 228.51, E_p = 8.9649$.	194.4 nm
Cody-Lorentz	$\epsilon_2(E) =$<br>$\begin{cases} \frac{E_g+E_t}{E} \cdot G(E_g+E_t) \cdot L(E_g+E_t) \cdot \exp\left(\frac{E-E_g-E_t}{E_u}\right) & (0 < E \leq E_g+E_t) \\ G(E) \cdot L(E) = \frac{(E-E_g)^2}{(E-E_g)^2+E_{pCL}^2} \cdot \frac{AE_o\Gamma E}{(E^2-E_o^2)^2+\Gamma^2E^2} & (E > E_g+E_t) \end{cases}$<br>$\epsilon_1(E) = 1 + \frac{A_p}{E_p^2-E^2} + \frac{2}{\pi}\mathcal{P}\int_0^\infty \frac{\xi\epsilon_2(\xi)}{\xi^2-E^2}d\xi$<br>$\epsilon_r = \epsilon_1 + i \cdot \epsilon_2$<br>where $A = 0.73147, E_o = 5.7237, \Gamma = 0.67386, E_g = 0.97139, E_{pCL} = 1, E_t = 0, E_u = 0.5, A_p = 229.12, E_p = 8.9752$.	194.4 nm

Note:
[a]Variables in blue in the ϵ_r column are the fitted parameters in each model.
[b]λ: free-space wavelength. All λ in this table have the unit of μm.
[c]E: photon energy. All E in this table have the unit of eV.
[d]i: all i in this table refer to imaginary unit ($i^2 = -1$).
[e]$\mathcal{P}$ denotes the Cauchy principal value.

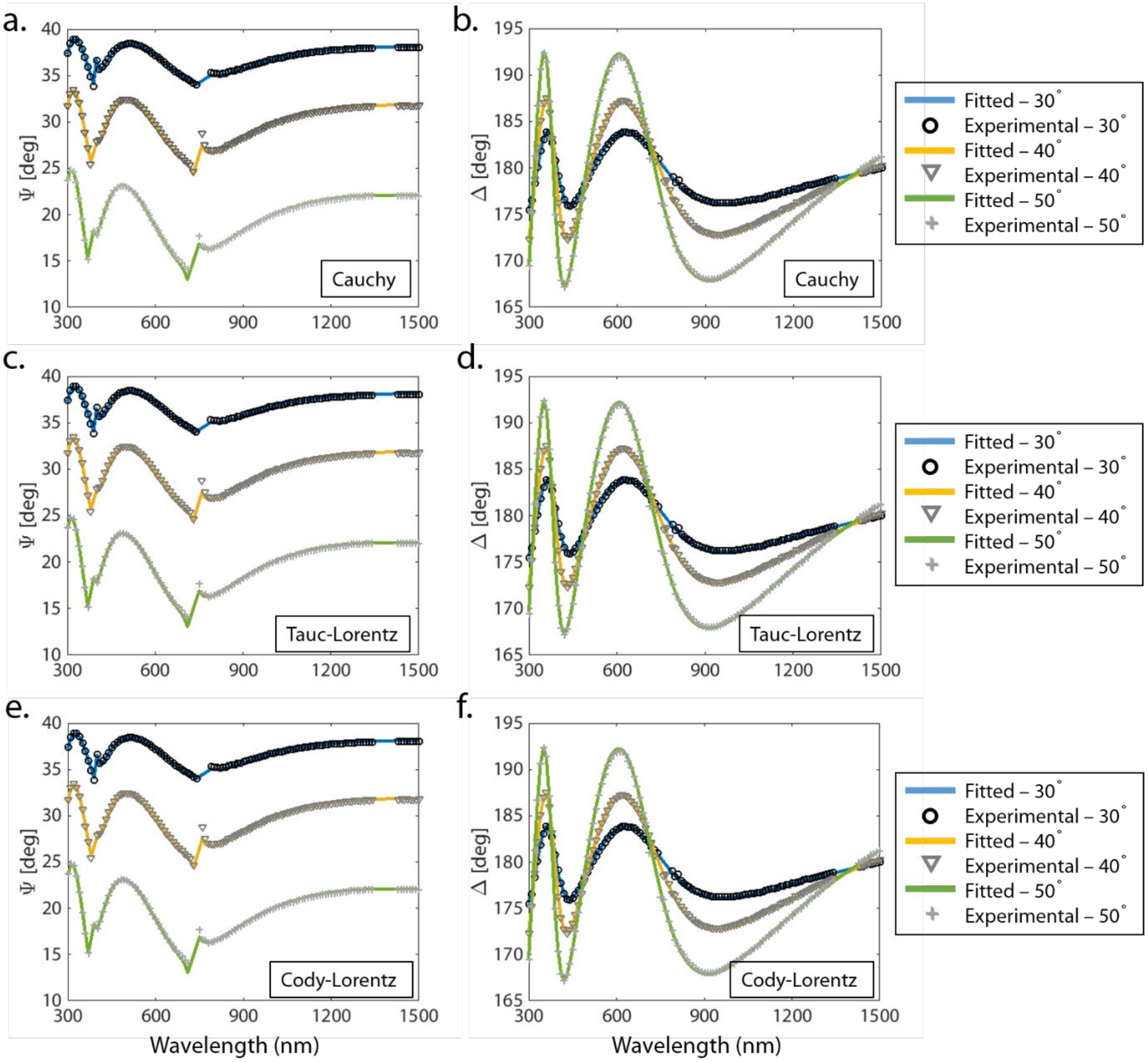

Fig. S2. Ψ and Δ data from the ellipsometry measurements at different incident angles, together with the model fitting results using the Cauchy model (a-b), the Tauc-Lorentz model (c-d), and the Cody-Lorentz model (e-f). All 3 models fit the experimental data well in this wavelength range.

We calculated the complex refractive indices from these 3 fitted models using the equation $n + i \cdot \kappa = \sqrt{\epsilon_r}$, where n is the real part of the refractive index, and κ is the extinction coefficient. Although those models agree on n values across the whole wavelength range, these models give κ values that differ by several orders of magnitude (**Fig. S3**). This is because our membrane is too thin and the extinction coefficient too small, leading to a very short optical path inside the membrane. Consequently, even a relatively big change in κ does not lead to a significant change in the ellipsometric parameters, and these findings necessitated the use of PCI to characterize the extinction coefficient of Si_3N_4. Furthermore, we note that all 3 models give $n = 2.004$ at 1064 nm for Si_3N_4, and the thickness of the membrane is ~194 nm. Those values were used in the PCI data analysis to determine the absorption coefficient of Si_3N_4 (see **Supplemental Information S5**).

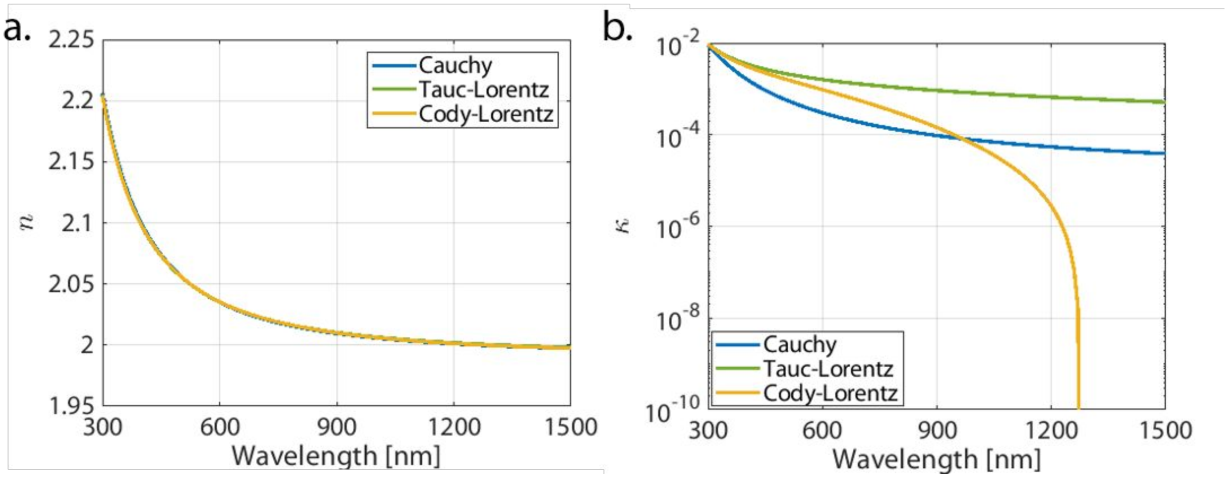

Fig. S3. Real (a) and imaginary (b) parts of the refractive indexes from fitted models. Although different models agree on n values, these models give different κ values, and therefore another characterization method with higher precision is needed to characterize κ for Si_3N_4.

We observed similar behavior for SiN_x ($x \sim 1$). Specifically, at 1064 nm, different models give $n = 2.147$, but give κ values that differ several orders of magnitude. All models show the thickness of the SiN_x membrane to be ~ 2.01 μm. Those values will be used in the PCI data analysis to determine the absorption coefficient of SiN_x (see **Supplemental Information S5**).

S4. Absorptivity measurements of reference samples using variable-angle spectroscopic ellipsometry

We determined the absorptivity of graphene-on-Si_3N_4 and graphene-on-SiN_x membranes using variable angle ellipsometry. Between samples with and without graphene, we observed enough difference in the ellipsometric parameters psi (Ψ) and delta (Δ) to conclude that graphene absorbance was observable via variable angle ellipsometry.

Si_3N_4

Our model to fit the ellipsometric data included a Si_3N_4 membrane coated by a 0.335 nm layer of graphite with a layer of water in between the two (**Fig. S4**). This is similar to the model used in Kravets et al.'s work on the ellipsometry of graphene[S11]; they fix the thickness of graphene to 0.335 nm and then fit the thickness of the water layer. **Figs. S4a** and **b** show the comparison of Ψ and Δ for samples with and without graphene; while measurements were taken at 3 different spatial positions on each sample at 65°, 70°, and 75° angles of incidence, data from only one measurement of each sample at 75° angle-of-incidence is shown in **Fig. S4** for clarity. A simple Drude oscillator model for the graphene also provided good fits, but we used a graphite model available in J.A. Woollam's WVASE library (**Table S3**) for its accuracy in capturing increasing absorption of graphene towards UV wavelengths[S11]. This fit resulted in a mean square error of fitting of ~1.1-2.3, and an absorptivity of 1.5% $\pm$ 0.11% for the graphene-on-Si_3N_4 sample at 1064 nm. We verified the accuracy of this model by noting that the absorptivity of the 0.335 nm layer of graphite alone was calculated to be 2.75% at 1064 nm, slightly higher than the well-known graphene absorbance of 2.3%.

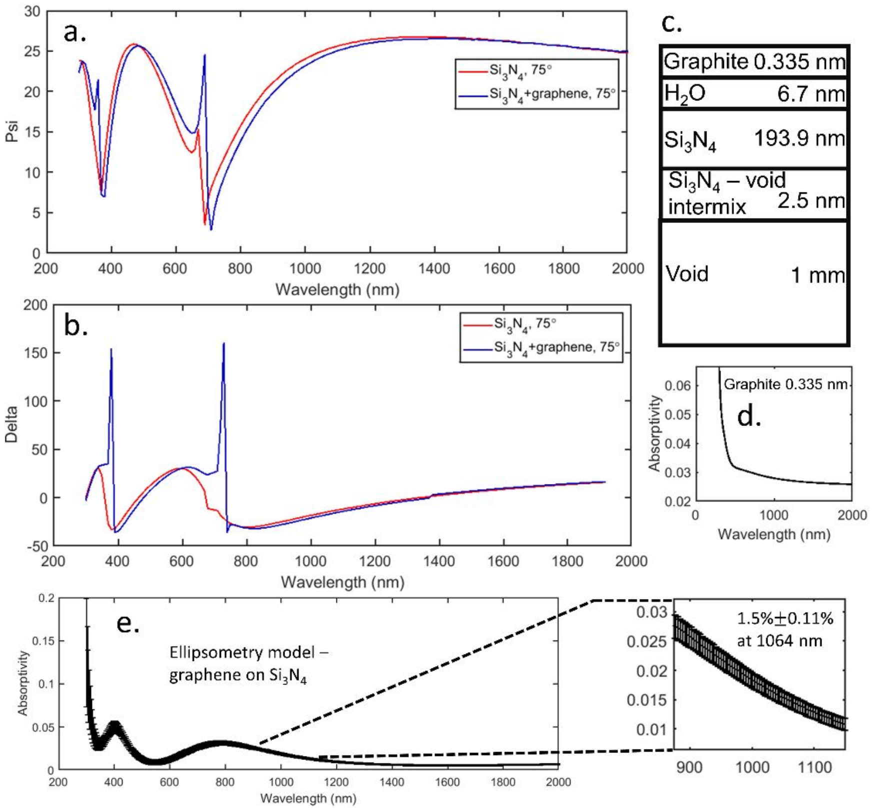

Fig. S4: Comparison of the measured (a) psi and (b) delta of Si_3N_4 membranes with and without graphene, (c) model used to fit ellipsometric data of samples with graphene, (d) calculated absorptivity of a 0.335 nm layer of graphite using a model from J.A. Woollam's library[S10], (e) calculated absorptivity of the model for the sample with graphene (1.5% $\pm$ 0.11%).

Table S3. Oscillators and parameters for the model describing graphite, used as a component of our model for graphene deposited on the Si_3N_4 membrane

Oscillator	Parameter and value		
Drude $\epsilon = -\frac{A_n Br_n}{E^2 + iBr_n E}$	A_n = 15.048	Br = 4.8914	
Gaussian $\epsilon = \epsilon_{n1} + i\epsilon_{n2}$ Where $\epsilon_{n2} = A_n e^{-\left(\frac{E-En}{\sigma}\right)^2} - A_n e^{-\left(\frac{E+En}{\sigma}\right)^2}$, $\epsilon_{n1} = \frac{2}{\pi}\mathcal{P}\int_0^\infty \frac{\xi\epsilon_{n2}(\xi)}{\xi^2-E^2}d\xi$, and $\sigma = \frac{Br_n}{2\sqrt{\ln(2)}}$	A_n = 2.8924	E_n = 2.0716	Br = 2.1145
Gaussian	A_n = 6.9623	E_n = 4.5182	Br = 0.8383
Gaussian	A_n = 2.5276	E_n = 5.2156	Br = 1.8065
Gaussian	A_n = 3.2927	E_n = 3.7064	Br = 1.2689

SiN_x

Our process to model graphene on SiN_x was similar to that with Si_3N_4 as detailed above, with the exception that SiN_x and graphene-on-SiN_x ellipsometry was done only at angles of 70°, 75° because the data at 65° was noisy. **Fig. S5** shows ellipsometric data comparison, model used and absorbance of the SiN_x membrane with graphene on it (2.6% ± 0.16% at 1064 nm).

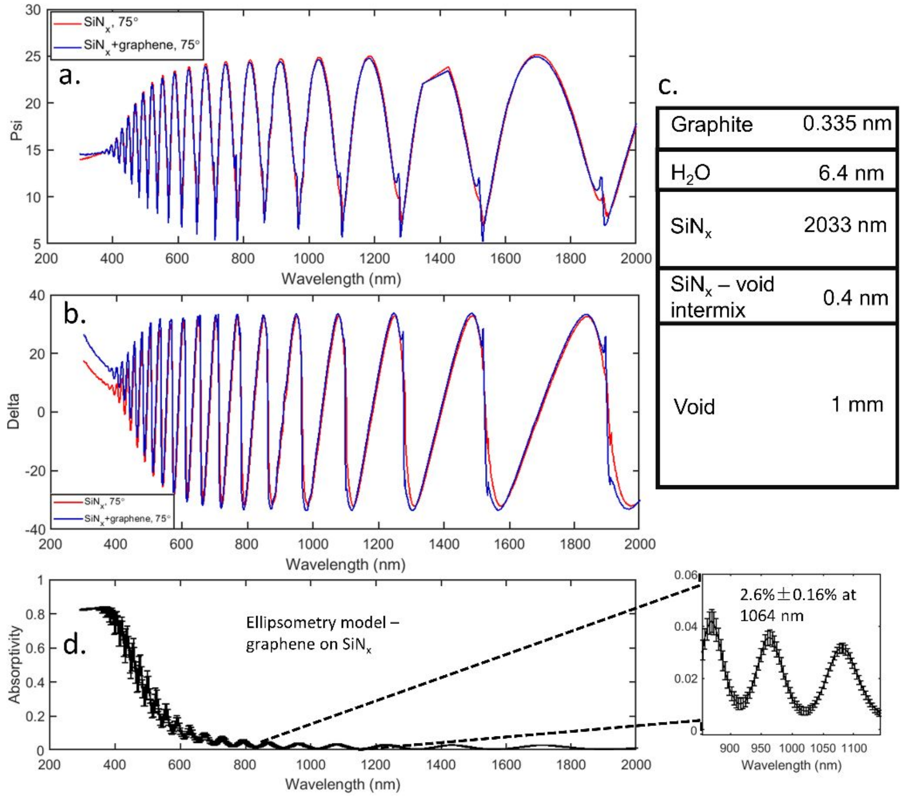

Fig. S5: Comparison of the measured (a) psi and (b) delta of SiN_x membranes with and without graphene, (c) model used to fit ellipsometric data of samples with graphene, (d) calculated absorptivity of the model for sample with graphene (2.6% ± 0.16% at 1064 nm).

S5. PCI data analysis

Membrane absorptivity calculations for Si_3N_4 (dataset 2) and SiNx

We used the self-referencing PCI method discussed in the main text to calculate the absorptivity of the Si_3N_4 and SiN_x membranes. **Eqns. (S1-2)** give the explicit equations we used to calculate the sample absorptivity

$$A^{ref} = K \cdot \frac{V_{AC}^{ref} \cdot P_{pump}^{ref}}{V_{DC}^{ref}} \tag{S1}$$

$$A^{SiN} = K \cdot \frac{V_{AC}^{SiN} \cdot P_{pump}^{SiN}}{V_{DC}^{SiN}} \tag{S2}$$

where A^{ref} (known via FTIR measurements) and A^{SiN} (to be determined) are the absorptivities of the reference and sample, respectively; K is the correction factor; V_{AC}^{ref} and V_{AC}^{SiN} are AC components of measured PCI signals for the reference and the sample, respectively; V_{DC}^{ref} and V_{DC}^{SiN} are DC components of

measured PCI signals for the reference and the sample, respectively; P_{pump}^{ref} and P_{pump}^{SiN} are pump-laser powers used in the PCI measurements (and measured with a power meter) for the reference and the sample, respectively. To convey an idea of the numbers involved, we show in **Table S4** data from a single position on the Si_3N_4 (dataset 2) and a single position on the SiN_x membrane (out of the 2601 points measured on each sample).

Table S4. Numerical values used to determine the absorptivity of one point each on the Si_3N_4 and SiN_x membranes

Si_3N_4 (dataset 2)		SiN_x	
A^{ref}	1.5%	A^{ref}	2.6%;
V_{AC}^{ref}	1.65E-4	V_{AC}^{ref}	0.03756
V_{DC}^{ref}	1.375	V_{DC}^{ref}	1.231
P_{pump}^{ref}	45.5 μW	P_{pump}^{ref}	20 mW
A^{sample}	3.38E-7	A^{sample}	1.94E-3
V_{AC}^{sample}	1.3E-4	V_{AC}^{sample}	0.0371
V_{DC}^{sample}	1.4763	V_{DC}^{sample}	1.248
P_{pump}^{SiN}	2 W	P_{pump}^{SiN}	253 mW
K	5.69E-3	K	1.70E-2

To determine the absorptivity of Si_3N_4 (dataset 2) and SiN_x membranes that we report in the main text, we conducted 2601 PCI measurements on both membranes over areas of 0.5 mm $\times$ 0.5 mm. Throughout those measurements, we kept P_{pump}^{sample} unchanged, and assumed K did not change with respect to different scanning locations for each type of membrane. We also found the variation of V_{DC}^{sample} to be negligible across different measurements. We recorded V_{AC}^{sample} for each measurement and used **Eqn. (S2)** to calculate the absorptivity for each scanning location. The results of the 2D scans are shown in **Fig. 3** in the main text, and histograms of those measured absorptivities for both Si_3N_4 (dataset 2) and SiN_x membranes are shown in **Fig. S7**.

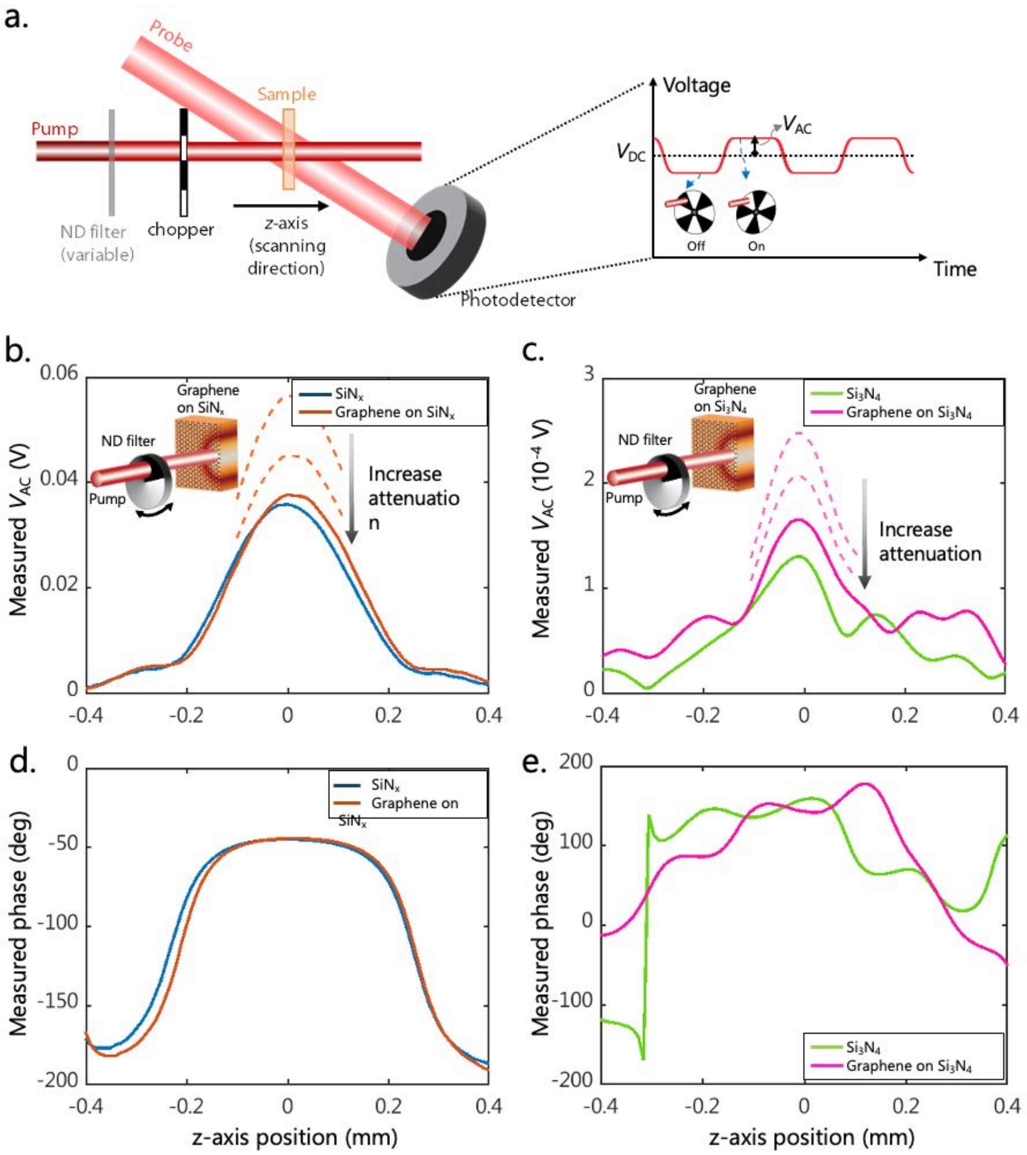

Fig. S6. Modified version of Fig. 2 from main text where panels (c) and (d) are using dataset 2 (rather than dataset 1, as in the main text). (a) Side-view schematic of the PCI setup showing translation of sample along the z-axis, and the AC and DC components of the detected signal. The sample is translated in the z-direction to find the peak of the AC signal which occurs when the pump waist is at the sample surface; (b) AC component of the detected probe intensity (V_{AC}) for the SiN_x membrane with and without graphene. The pump intensity was attenuated using a variable ND-filter for the sample with graphene to obtain a similar V_{AC} to that of SiN_x alone (inset). Solid lines are the measured V_{AC}, while dashed lines represent the process of increasing attenuation to achieve similar V_{AC} with and without graphene; (c) V_{AC} for the Si_3N_4 (dataset 2) membrane with and without graphene, similarly obtained by attenuation using a variable ND-filter; (d, e) Phase between the chopped pump and detected probe intensities vs. the sample position for (d) the SiN_x membrane and (e) Si_3N_4 membrane, with and without graphene.

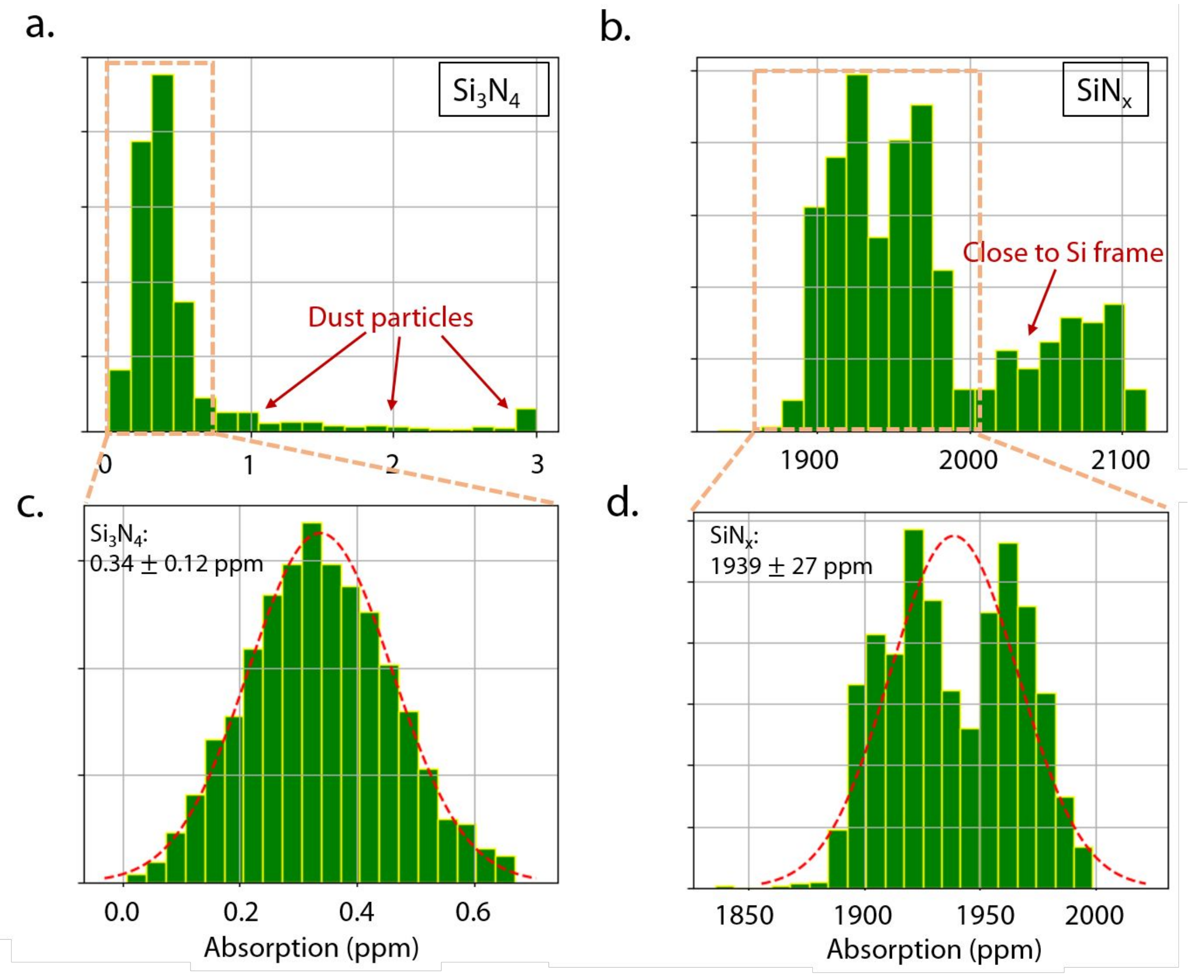

Fig. S7. (a-b) Histograms of 2D scans of absorptivity for (a) Si_3N_4 (dataset 2), and (b) SiN_x ($x \sim 1$) membranes, in parts per million (ppm). Note that for SiN_x there is a bimodal distribution, with the higher-absorption points due to proximity to the Si frame. (c) Histogram and a Gaussian fit of the absorptivity of Si_3N_4 (dataset 2), after removing data points corresponding to dust particles. (d) Histogram and a Gaussian fit of the absorptivity of SiN_x ($x \sim 1$), after removing data points that are close to Si frame. In these figures, we assume A^{ref} = 2.6 % for graphene-on-SiN_x reference, and A^{ref} = 1.5 % for graphene-on-Si_3N_4 reference.

As we discussed in the manuscript, for the Si_3N_4 (dataset 2), dust particles lead to absorption peaks in PCI measurements, and in **Fig. S7a** they correspond to the long-tail feature in the histogram. To remove those points, we discarded all data points with absorptivity > 0.67 ppm, and did a Gaussian fit on the remaining data points (**Fig. S7c**). This gives us an absorptivity of $(3.4 \pm 1.2) \times 10^{-7}$ for the Si_3N_4 (dataset 2). For the SiN_x membrane, we observed an increase in absorptivity when the pump laser beam was close to the Si frame, and this leads to a double-peak pattern in the histogram (**Fig. S7b**). To remove those points, we discarded all data points with absorptivity > 2000 ppm, and did a Gaussian fit on the remaining data points (**Fig. S7d**). This gives us an absorptivity of $(1.94 \pm\ 0.027) \times 10^{-3}$ for the SiN_x membrane. Note that in this paragraph, the uncertainty of the absorptivity estimate comes directly from the distributions in **Fig. S7**, and may underestimate the true uncertainty, as described in the next section.

Error-bar calculations for Si_3N_4 (dataset 2) and SiNx

In **Fig. S7**, the distribution of absorptivity values is indicative of uncertainty due to variation in the material across the sample and noise in the PCI setup. However, for us to appropriately estimate the absorption coefficient, we also need to consider the uncertainty of the measured absorptivity values of reference samples (A^{ref}). Here, we assume these 2 sources of uncertainty are independent.

As described in the main text, the absorption of the reference (graphene-coated SiN membrane), A^{ref}, is $(1.5 \pm 0.11)\%$ for graphene-on-Si_3N_4 and $(2.6 \pm 0.16)\%$ for graphene-on-SiN_x. To translate the uncertainty of A^{ref} to the uncertainty of absorptivity, we repeat the process in the above subsection, with $A^{ref} = (1.5 - 0.11)\%, 1.5\%, (1.5 + 0.11)\%$ for graphene-on-Si_3N_4 reference, and $A^{ref} = (2.6 - 0.16)\%, 2.6\%, (2.6 + 0.16)\%$ for graphene-on-SiN_x reference, and calculate the standard deviations of the absorptivities of Si_3N_4 (dataset 2) and SiN_x with those different A^{ref} values (denoted as $\sigma_A^{Si_3N_4}$ and $\sigma_A^{SiN_x}$, respectively).

The total error bar of absorptivity, $\sigma_{total}^{Si_3N_4}$ and $\sigma_{total}^{SiN_x}$, can be estimated using equations $\sigma_{total}^{Si_3N_4} = \sqrt{\left(\sigma_A^{Si_3N_4}\right)^2 + \left(\sigma_{PCI}^{Si_3N_4}\right)^2}$, $\sigma_{total}^{SiN_x} = \sqrt{\left(\sigma_A^{SiN_x}\right)^2 + \left(\sigma_{PCI}^{SiN_x}\right)^2}$, where $\sigma_{PCI}^{Si_3N_4}$ and $\sigma_{PCI}^{SiN_x}$ are standard deviations from PCI measurements calculated in the previous subsection (1.2×10^{-7} for Si_3N_4, and 0.027×10^{-3} for SiN_x). Using this approach, we obtained the absorptivity of Si_3N_4 (dataset 2) to be $(3.4 \pm 1.23) \times 10^{-7}$, and the absorptivity of SiN_x membrane to be $(1.94 \pm 0.12) \times 10^{-3}$.

Absorption-coefficient calculations for Si_3N_4 (dataset 2) and SiNx

To convert the absorptivities of the membranes to the absorption coefficients of Si_3N_4 (dataset 2) and SiN_x, we applied the transfer-matrix method[S12] on an infinitely wide single-layer membrane surrounded by air as illustrated in **Fig. S8**. For the Si_3N_4 (dataset 2), the thickness used was 194.4 nm (fitted from ellipsometry, **Table S2**), and the complex refractive index at 1064 nm is $2.004 + \frac{\alpha_{Si_3N_4} \cdot \lambda}{4\pi} \cdot i$, where $\lambda = 1064\, nm$, and $\alpha_{Si_3N_4}$ is the unknown to be solved. Using the root finder, we found when $\alpha_{Si_3N_4} = (2.09 \pm 0.76) \times 10^{-2}$ cm^{-1}, the membrane had an absorptivity of $(3.4 \pm 1.23) \times 10^{-7}$ (corresponding to the PCI measurement result). Similarly, for the SiN_x membrane, the thickness used was 2.01 μm, and the complex refractive index used at 1064 nm was $2.147 + \frac{\alpha_{SiN_x} \cdot \lambda}{4\pi} \cdot i$, where $\lambda = 1064\, nm$, and α_{SiN_x} was the unknown to be solved. Using the root finder, we found when $\alpha_{SiN_x} = 7.94 \pm 0.50$ cm^{-1}, the absorptivity of the membrane was the same as our PCI measurement result ($(1.94 \pm 0.12) \times 10^{-3}$).

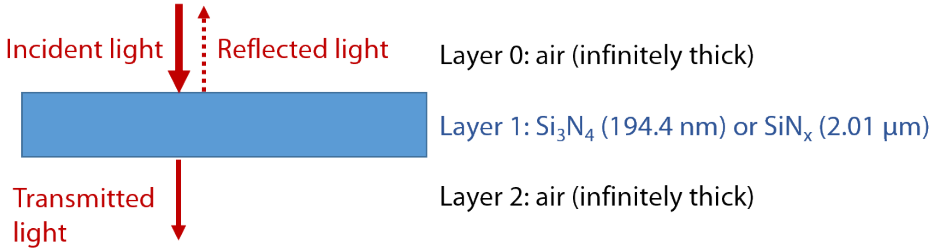

Fig. S8. Illustration of the model used in the transfer-matrix calculation to determine the absorption coefficients of Si_3N_4 and SiN_x.

Response of V_{AC} and phase to pump power

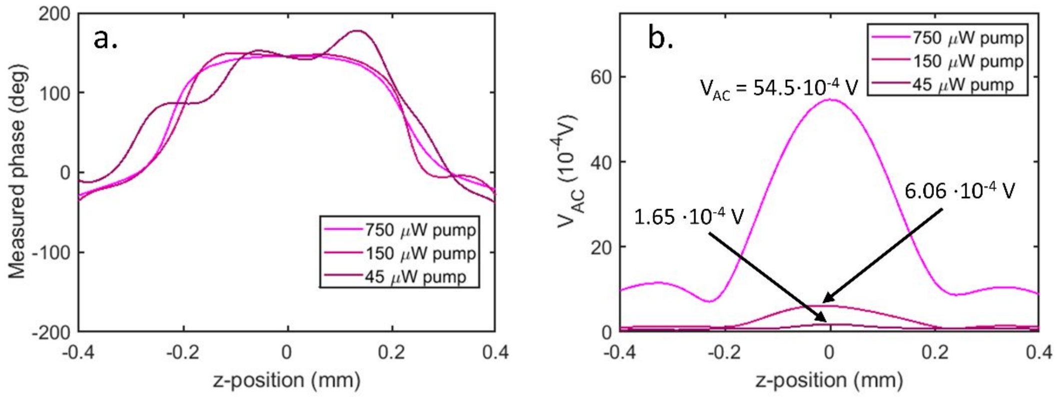

Fig. S9 (a) Phase vs z-position plot for the PCI scan of Si_3N_4 coated with monolayer graphene (dataset 2) at different pump powers. The phase is independent of the pump power, although the trend becomes noisier for low pump powers (45 μW in this case) due to low signal-to-noise ratio. (b) V_{AC} for different pump powers. In our self-referencing PCI experiment, the pump was manually attenuated to ~45 μW for Si_3N_4-with-graphene to achieve a V_{AC} close to that of bare Si_3N_4 with pump power of 2 W. We note that **Fig. S9** is based on data from dataset 2 of graphene-coated Si_3N_4, which is why the V_{AC} and phase numbers are different from what is in the current version of the main text in **Fig. 2**—where dataset 1 is used.

Fig. S9 shows how phase and V_{AC} change as the pump power is increased more than an order of magnitude from ~45 μW to ~750 μW for graphene on Si_3N_4. **Fig. S9(a)** shows that the phase is largely independent of pump power, with the signal becoming noisier at the lowest pump power of ~45 μW. **Fig. S9(b)** shows how the V_{AC} to pump-power ratio changes for a range of powers across more than an order of magnitude. **Table S5** below summarizes data from **Fig. S9(b)**:

Table S5. V_{AC} and V_{AC} to pump-power ratio for a graphene on Si_3N_4 sample

Pump power (μW)	V_{AC} (10^{-4}V)	V_{AC} to pump ratio (10^{-6}V/μW)
45	1.65	3.7
150	6.06	4
750	54.5	7.3

We note that the V_{AC} to pump power ratio increases and therefore the photothermal effect as a function of pump is non-linear as pump power is increased more than an order of magnitude (shown for graphene on Si_3N_4 in **Table S5**). However, by design this is not a problem in self-referencing PCI, because we conduct experiments at similar V_{AC} between the reference sample and the sample being tested (graphene-Si_3N_4 and Si_3N_4 in this work), which corresponds to similar amounts of photothermal lensing. In this way, self-referencing PCI ensures that we always operate in quasi-linear regimes.

S6. Comparison of measured loss in Si_3N_4 with other works

Table S6. Loss and absorption coefficient of Si_3N_4 reported in other papers

Work	Method	Growth	Wavelength (nm)	Loss in dB cm^{-1}	Absorption coefficient (cm^{-1})
Our work	Self-referencing PCI	LPCVD (purchased from Norcada Inc.)	**1064**	5.43×10^{-2}	~1.25×10^{-2}
Land/Wilson 2024[S13]	Nanomechanical absorption spectroscopy	LPCVD	**532, 633, 750, 780, 800, 850, 1064, 1550**	1.36, 0.63, 0.34, 0.29, 0.26, 0.23, 0.09, 0.24	0.31, 0.15, 0.08, 0.07, 0.06, 0.05, 0.02, 0.06
Ikeda/ Fainman 2008[S14]	Measure loss vs different waveguide lengths	PECVD	**1548**	4 (transmission loss)	0.92
Ji/ Lipson 2017[S15]	Cavity ringdown; compare losses in different structures	LPCVD	**1560**	(1.3 ± 0.5) ×10^{-3} (bulk)	~3×10^{-4}
Luke/ Lipson 2013[S16]	α calculated from Q factor	LPCVD	**1550**	4.2×10^{-2} (transmission loss); 2.94×10^{-2} (absorption loss)	9.7×10^{-3} (transmission loss); 6.8×10^{-3} (absorption loss)

S7. Application in light sails

Here we calculate the equilibrium temperature of a hypothetical light sail under laser illumination, modeled as an infinitely-wide thin membrane (**Fig. S10(a)**). The membrane thicknesses of such hypothetical light-sails are chosen such that the reflectivities of the sails are maximized. Using the transfer-matrix method, we determined the optimal thickness of Si_3N_4-based light sail to be 132.68 nm with a reflectivity of 0.36, and the optimal thickness of SiN_x-based light sail to be 123.57 nm with a reflectivity of 0.42.

In a sail under illumination, two major thermal processes happen. First, the sail absorbs incident light due to its non-zero absorption coefficient, leading to an increase in the temperature. Second, the sail emits electromagnetic waves that lead to cooling (known as radiative cooling) (**Fig. S10(a)**). The equilibrium temperature T_{eq} is reached when the power absorbed is equal to the power emitted and, to maintain sail integrity, the upper limit of T_{eq} should be lower than the ultra-high vacuum (UHV) melting temperature of Si_3N_4[S17] Here we set the upper limit of T_{eq} to be 1500 K, which is on the lower end of the estimates of the decomposition temperature of Si_3N_4[S18,S19].

We thus calculated the equilibrium temperature T_{eq} reached by each sail using Eqn. S3:

$$A \cdot P_{inc} = 2A_{sail} \int_a^b \frac{c_1}{\lambda^5} \cdot \frac{\epsilon_{sail}(\lambda)}{\exp\left(\frac{c_2}{\lambda \cdot T_{eq}} - 1\right)} d\lambda \qquad \text{(S3).}$$

The left-hand side is power absorbed by the sail (P_{abs}), and the right-hand side is emitted black-body radiation (P_{em}), where A is the absorbance of the sail, P_{inc} is the incident laser power on the sail, A_{sail} is the area of a single side of the sail, the factor of 2 accounts for the emission on both sides of the sail, $a = 1.4\ \mu m$ and $b = 32\ \mu m$ are the integration bounds that capture the vast majority of thermal radiation and are the bounds for SiN material properties from Luke et al.[S20], $\epsilon_{sail}(\lambda)$ is the wavelength-dependent spectral emissivity of the sail, $c_1 = 2\pi hc^2$, $c_2 = hc/k_b$, h is Planck's constant, c is the speed of light, and k_b is the Boltzmann's constant. Here, A for a given sail geometry is calculated using the transfer-matrix method and the absorption coefficient measured by PCI. $\epsilon_{sail}(\lambda)$ for a given sail is calculated using the transfer-matrix method and the complex refractive index of Si_3N_4[S12]. For simplicity, we assume $\epsilon_{sail}(\lambda)$ for SiN_x to be the same as that for Si_3N_4.

The absorbed and emitted power densities under 10 GW/m^2 of illumination (which is the driving laser power density in the Breakthrough Starshot mission)[S21] as a function of temperature are plotted in **Fig. S10(b)** for Si_3N_4, and in **Fig. S10(c)** for SiN_x. The temperature at which $P_{abs} = P_{em}$ is the equilibrium temperature T_{eq}. For our highest reported Si_3N_4 absorbance of 3 × 10^{-2} cm^{-1}, we observed the equilibrium temperature T_{eq} of the Si_3N_4 sail to be approximately 940 K, lower than the decomposition temperature (1500 K) of Si_3N_4. It must be noted however that the calculated equilibrium temperature will likely change with the use of temperature-dependent absorption data and more optimized sail designs[S21,S22]. We note that for SiN_x, an equilibrium temperature is not achieved at the GW/m^2 power scale. We also calculated the equilibrium temperature as a function of incident laser intensity. To achieve a similar equilibrium temperature for SiN_x as for Si_3N_4, the laser power must be ~3 orders of magnitude lower (**Fig. S10(d)**).

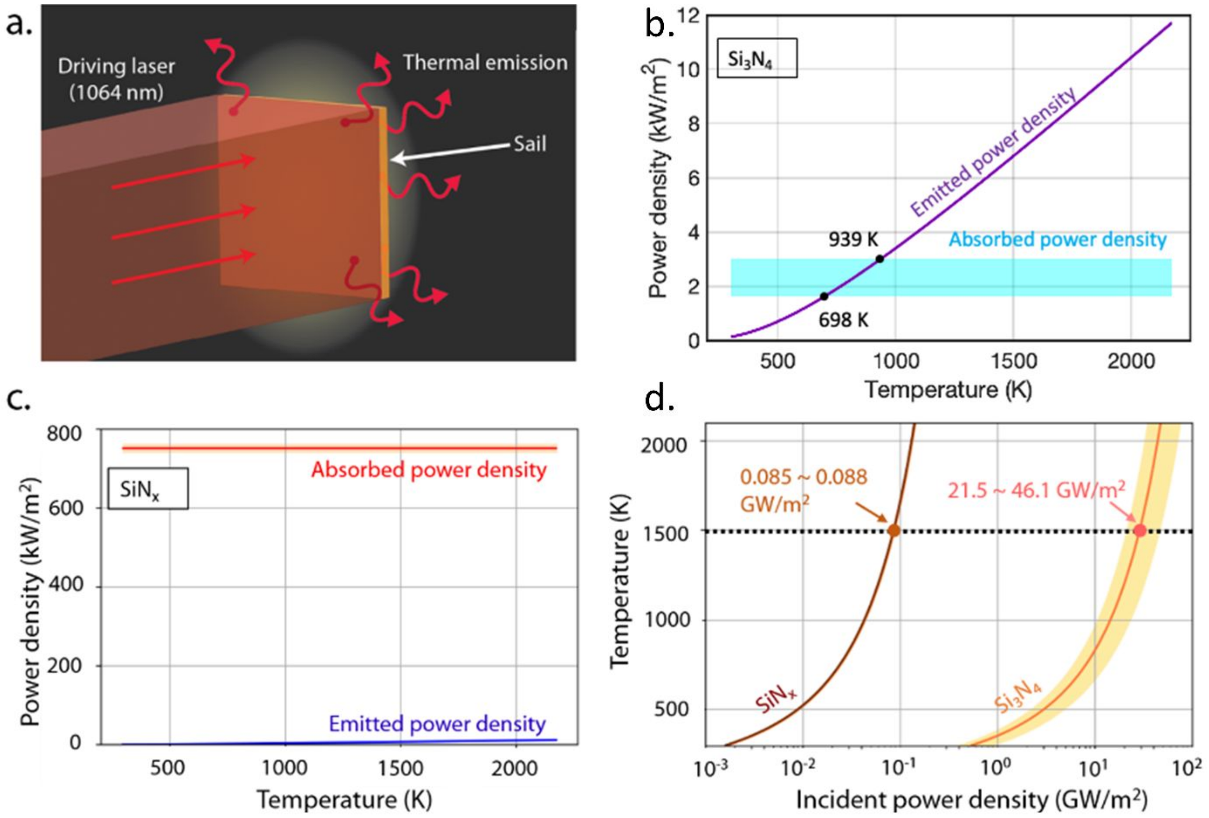

Fig. S10. (a) Schematic of a silicon-nitride sail in space illuminated by a 1064 nm laser used in our thermal-equilibrium calculation. In our calculation, both the silicon-nitride sail and the incident laser are infinitely wide, and the incident laser has a uniform power density. (b) Absorbed and emitted power density vs sail temperature for a Si_3N_4 sail, illuminated by a 10 GW/m^2 laser with $\lambda = 1064$ nm. The shaded cyan region represents absorbed power density for the absorptivity range of Si_3N_4 presented in the main text. The marked temperatures are points of equilibrium for lowest and highest calculated absorptivities, where absorbed and emitted power densities are equal. (c) Absorbed and emitted power density vs sail temperature for a SiN_x sail, illuminated by a 10 GW/m^2 laser with $\lambda = 1064$ nm. The orange shaded region corresponds to the error bar of the absorbed power density, due to the uncertainty of absorption coefficient of SiN_x. No thermal equilibrium can be reached within the 300-2100 K temperature range for this SiN_x sail. (d) Equilibrium temperature as a function of incident laser intensity for the Si_3N_4 sail and the SiN_x sail considered in (b) and (c). The dashed line corresponds to the decomposition temperature of Si_3N_4 at 1500 K. Shaded regions correspond to error bars of incident laser intensity, due to the uncertainty of absorption coefficient of Si_3N_4 and SiN_x.

S8. Summary of experimental methods

Sample details

We measured the absorptivity in ~194-nm thick Si_3N_4 and ~2-µm thick SiN_x membranes (purchased from Norcada Inc., Edmonton, AB, Canada), suspended on 200 µm thick silicon frames. The thicknesses of the membranes were calculated from ellipsometric measurements. In the in-plane direction, membranes of both stoichiometries had the same dimensions with a silicon frame of 10 mm x 10 mm and a freestanding membrane area of 5 mm x 5 mm (see **Supplemental Information S2**).

PCI experimental setup

The PCI setup comprised a 1064 nm pump laser (YLR-10-LP, IPG Photonics) and a 633 nm probe laser (JDSU 1122P HeNe laser). The pump beam was chopped at ~390 Hz and its power measured by a thermopile detector (Thorlabs S310C), and the modulated probe signal measured with a detector (DET10A Si detector, Thorlabs, Inc.), connected to a lock-in amplifier (SRS SR810) (**Fig. 1a** in main text).

The pump and probe beams were set to cross each other at their beam waists. The sample was moved in the z-direction until its surface was in the sample plane as the beam waists, indicated by a characteristic peak in the AC signal[S23] (**Fig. 2** in main text). All subsequent measurements for a given sample were conducted at the z-position thus obtained.

To obtain similar AC voltage values for the graphene-coated reference and the sample to be measured, the pump laser power was appropriately attenuated for the former. In normal PCI operation the pump is attenuated with a half-wave plate and polarizer; for powers lower than 1 mW, we used an additional neutral density (ND) filter with optical density (OD) of 0.9. In this power regime, a power meter (Thorlabs S130VC) was placed between the chopper and the sample to note the power. 1-D z-scan (longitudinal direction) PCI signals (**Fig. 2** in main text) as well as 0.5 mm × 0.5 mm 2D maps were acquired for each sample (**Fig. 3** in main text).

For each stoichiometry, upon adjusting the pump for similar AC signal values with and without graphene, we noted the ratio of the respective pump powers required, to calculate absorptivity values. To account for surface variations, we measured the PCI signal at multiple spatial points on each membrane. We conducted measurements at 5 points on Si_3N_4 (dataset 1) and at 2601 points for Si_3N_4 (dataset 2). For SiN_x, a single data set of 2601 points sufficed; a relatively good signal was measured because of the higher absorptivity of SiN_x. We discarded anomalous data such as possible specks of dust and increasing absorptivity close to the frame.

Transfer of graphene onto SiN membranes, and ellipsometry characterization

Graphene was transferred onto the Si_3N_4 and SiN_x membranes using a wet transfer method. Polymethyl methacrylate (PMMA) was spin-coated onto CVD graphene grown on a copper foil (obtained from Grolltex Inc., San Diego, CA). The Cu foil was etched away in $FeCl_3$. The graphene was then transferred onto the membranes and baked at 60 °C to ensure good adhesion and the removal of water between graphene and the membrane. The PMMA was removed by an acetone bath at 60 °C.

The absorptivities of the graphene-coated Si_3N_4 and SiN_x membranes were calculated using variable angle spectroscopic ellipsometry (J.A. Woollam V-VASE). First, the thickness and refractive index (n) of the Si_3N_4 and SiN_x membranes were obtained from models fitted to the ellipsometric parameters psi Ψ and delta Δ. Then, these models were used as substrates for ellipsometric data of samples with graphene, and their absorptivities calculated using J.A. Woollam's WVASE software (which uses the transfer matrix method). **Supplemental sections S3 and S4** list out more details about the ellipsometry and corresponding fitting.

Frequency-domain thermoreflectance measurements of Si_3N_4 and SiN_x thermal conductivity

We used frequency-domain thermoreflectance (FDTR) to measure the thermal conductivities of the SiN_x and Si_3N_4 membranes. We deposited a ~100 nm Au film, which has a large temperature coefficient of thermoreflectance[S24], on the membranes using electron beam evaporation. The pump (488 nm, Coherent Genesis MX 1W) and probe (532 nm Coherent OBIS LX 20 mW) beams were focused through a 20 × infinity-corrected objective lens to achieve 6.3 and 5.9 μm spot sizes at the transducer surface. The pump and probe powers were fixed at 3 mW and 2.2 mW, respectively, in order to limit the temperature-rise at the sample surface to < 1 K[S25]. Literature values for the volumetric heat capacities of SiN_x[S26] and Si_3N_4[S27] were used for extracting the thermal boundary conductance at the Au/membrane interfaces and the in-plane thermal conductivities of the membranes. Using FDTR, we fit both the in-plane and cross-plane thermal conductivities of thin-film samples. In our FDTR measurements, we were not sensitive to the cross-plane thermal conductivity of the membrane - the thermal penetration depth of the heating laser was larger than the membrane thicknesses for a significant part of the applied frequency range, causing the response of the probe beam to be governed by only the in-plane thermal transport. The cross-plane thermal conductivities were measured separately in regions where the membrane is supported by a silicon substrate, and confirmed that we were not sensitive to this value in regions where the membrane was suspended.

S9. References

[S1] K. V. Vlasova, A. I. Makarov, N. F. Andreev, High-sensitive absorption measurement in ultrapure quartz glasses and crystals using time-resolved photothermal common-pass interferometry and its possible prospects. *J. Appl. Phys.* **2021**, *129*, 4, DOI: 10.1063/5.0020437.

[S2] K. V Vlasova, A. I. Makarov, N. F. Andreev, A. Y. Konstantinov, High-sensitive absorption measurement in transparent isotropic dielectrics with time-resolved photothermal common-path interferometry. *Appl. Opt.* **2018**, *57*, 22, 6318-6328.

[S3] M. Marchiò, Development of an optical absorption measurement system to characterize KAGRA sapphire mirrors and new high-reflectivity crystalline coatings, *Ph.D. Thesis*, University of Tokyo, December, **2018**, https://repository.dl.itc.u-tokyo.ac.jp/record/2001880/files/A35712.pdf (accessed 2025-10-09).

[S4] R. Birney, J. Steinlechner, Z. Tornasi, S. Macfoy, D. Vine, A. S. Bell, D. Gibson, J. Hough, S. Rowan, P. Sortais, S. Sproules, S. Tait, I. W. Martin, S. Reid, *Phys. Rev. Lett.* **2018**, *121*, 191101, DOI: 10.1103/PhysRevLett.121.191101.

[S5] D.-S. Tsai, Z.-L. Huang, W.-C. Chang, S. Chao, Amorphous silicon nitride deposited by an NH_3 - free plasma enhanced chemical vapor deposition method for the coatings of the next generation laser interferometer gravitational waves detector. *Classical Quantum Gravity* **2022**, *39*, 15LT01, DOI: 10.1088/1361-6382/ac79f6.

[S6] H.-W. Pan, L.-C. Kuo, S.-Y. Huang, M.-Y. Wu, Y.-H. Juang, C.-W. Lee, S. Chao, Silicon-nitride Films Deposited by PECVD Method on Silicon Substrate for Next Generation Laser Interference Gravitational Wave Detector. In *Optical Coatings for Gravitational Wave Detection*, Optical Interference Coatings, Tucson, **2016**, DOI: 10.1364/OIC.2016.MB.12.

[S7] J. Steinlechner, I. W Martin, A. Bell, G. Cole, J. Hough, S. Penn, S. Rowan, S. Steinlechner, Mapping the optical absorption of a substrate-transferred crystalline AlGaAs coating at 1.5 μm. *Classical Quantum Gravity* **2015**, *32*, 105008, DOI: 10.1088/0264-9381/32/10/105008

[S8] Y.-J. Lee, A. Das, M. L. Mah, J. J. Talghader, Long-wave infrared absorption measurement of undoped germanium using photothermal common-path interferometry. *Appl. Opt.* **2020**, *59*, 3494-3497.

[S9] M. Leidinger, S. Fieberg, N. Waasem, F. Kühnemann, K. Buse, I. Breunig, *Opt. Express* **2015**, *23*, 21690-21705.

[S10] J.A. Woollam Co. Inc., Guide to Using WVASE, accessed: March **2024**.

[S11] V. G. Kravets, A. N. Grigorenko, R. R. Nair, P. Blake, S. Anissimova, K. S. Novoselov, A. K. Geim, Spectroscopic ellipsometry of graphene and an exciton-shifted van Hove peak in absorption. *Phys. Rev. B* **2010**, *81*, 155413, DOI: 10.1103/PhysRevB.81.155413.

[S12] S. J. Byrnes, (Preprint) arXiv: 1603.02720 v5, submitted: Dec **2020**.

[S13] A.T. Land, M. Dey Chowdhury, A.R. Agrawal, D.J. Wilson, Sub-ppm Nanomechanical Absorption Spectroscopy of Silicon Nitride. *Nano Lett.* **2024**, *24*, 25, 7578-7583.

[S14] K. Ikeda, R. E. Saperstein, N. Alic, Y. Fainman, Thermal and Kerr nonlinear properties of plasma-deposited silicon nitride/ silicon dioxide waveguides. *Opt. Express* **2008**, *16*, 12987-12994.

[S15] X. Ji, F. A. S. Barbosa, S. P. Roberts, A. Dutt, J. Cardenas, Y. Okawachi, A. Bryant, A. L. Gaeta, M. Lipson, *Optica* **2017**, *4*, 6, 619-624.

[S16] K. Luke, A. Dutt, C. B. Poitras, M. Lipson, Overcoming Si_3N_4 film stress limitations for high quality factor ring resonators. *Opt. Express* **2013**, *21*, 22829-22833.

[S17] J. Brewer, M. F. Campbell, P. Kumar, S. Kulkarni, D. Jariwala, I. Bargatin, A. P. Raman, Multiscale Photonic Emissivity Engineering for Relativistic Lightsail Thermal Regulation. *Nano Lett.* **2022,** *22*, 2, 594-601.

[S18] R. C. Brown, P. K. Swaminathan, Silicon nitride equation of state. *AIP Conf. Proc* **2017**, *1793*, 1, 050013, DOI: 10.1063/1.4971547.

[S19] H. D. Batha, E. D. Whitney, Kinetics and Mechanism of the Thermal Decomposition of Si_3N_4. *J. Am. Ceram. Soc.* **1973**, *56*, 365-369.

[S20] K. Luke, Y. Okawachi, M. R. E. Lamont, A. L. Gaeta, M. Lipson, Broadband mid-infrared frequency comb generation in a Si3N4 microresonator., *Opt. Lett.* **2015**, *40*, 4823-4826.

[S21] H. A. Atwater, A. R. Davoyan, O. Ilic, D. Jariwala, M. C. Sherrott, C. M. Went, W. S. Whitney, J. Wong, Materials challenges for the Starshot lightsail. *Nat. Mat.* **2018**, *17*, 861-867.

[S22] G. R. Holdman, G. R. Jaffe, D. Feng, M. S. Jang, M. A. Kats, V. W. Brar, Thermal Runaway of Silicon-Based Laser Sails. *Adv. Opt. Mater.* **2022**, *10*, 2102835, DOI: 10.1002/adom.202102835.

[S23] A. Alexandrovski, M. Fejer, A. Markosian, R. Route, Photothermal common-path interferometry (PCI): new developments. In *Solid State Lasers XVIII: Technology and Devices* (Eds.: W. A. Clarkson, N. Hodgson, R. K. Shori), Vol. 7193, pp. 79-91, SPIE, San Jose **2009**.

[S24] J. Christofferson, A. Shakouri, Thermoreflectance based thermal microscope. *Rev. Sci. Instrum.* **2005**, *76*, 024903, DOI: 10.1063/1.1850632.

[S25] J. L. Braun, C. J. Szwejkowski, A. Giri, P. E. Hopkins, On the Steady-State Temperature Rise During Laser Heating of Multilayer Thin Films in Optical Pump-Probe Techniques. J. Heat Transfer **2018**, *140*, 5, DOI: 10.1115/1.4038713

[S26] E. Franke, D. A. Lavan, C. A. Volkert, Quantifying DC differential scanning nanocalorimetry for determining heat capacities. *Thermochim. Acta* **2018**, *668*, 116-125.

[S27] K. Watari, K. Hirao, M. Toriyama, K. Ishizaki, Effect of grain size on the thermal conductivity of Si_3N_4. *J. Am. Ceram. Soc.* **1999**, *82*, 777-779.
